# Supplementary material for: Attitude and beliefs about the social environment associated with chemsex among MSM visiting STI clinics in the Netherlands: An observational study
Source: PLoS One. 2020 Jul 1;15(7):e0235467. doi: 10.1371/journal.pone.0235467 (PMC7329118; doi:10.1371/journal.pone.0235467)
Supplement: S1 File — (DOCX) [file pone.0235467.s001.docx]

**S1 File Informed consent in Dutch and English**

Beste meneer,

Wij vragen u om mee te doen aan een onderzoek van de GGD om de zorg en SOA bestrijding te verbeteren. U ontvangt deze vragenlijst omdat u tijdens uw bezoek hebt aangegeven dat we u mochten uitnodigen voor dit onderzoek. Voordat u beslist of u wilt meedoen aan dit onderzoek, krijgt u uitleg over wat het onderzoek inhoudt. Lees deze informatie rustig door en vraag de onderzoeker uitleg als u vragen heeft.

**Achtergrondinformatie**

Momenteel krijgt de GGD regelmatig vragen van bezoekers over drugs. We willen onze kennis hierover verbeteren om zorg te kunnen leveren die aansluit bij de wensen en behoeften van bezoekers. Doelen van dit onderzoek zijn om:

• te onderzoeken hoe vaak drugsgebruik tijdens seks voorkomt;

• de context van drugsgebruik tijdens seks in kaart te brengen;

• de relatie tussen drugsgebruik en SOA te onderzoeken;

• de behoeften aan professionele hulp te identificeren.

Ook als u geen ervaring hebt met drugs, is uw deelname belangrijk om de onderzoeksvragen te kunnen beantwoorden.

**Wat houdt meedoen in?**

Meedoen aan dit onderzoek betekent dat u een vragenlijst invult van ongeveer 20 minuten. De antwoorden op de vragenlijst worden alleen gebruikt voor onderzoeksdoeleinden. De antwoorden zijn niet zichtbaar voor de SOA verpleegkundigen, doktersassistenten of artsen. Deelname heeft geen invloed op de zorg die u ontvangt bij de GGD. De vragenlijstgegevens worden gekoppeld aan enkele gegevens uit het meest recente SOA consult op de GGD, zoals testuitslagen, postcode, etniciteit, leeftijd en opleidingsniveau. Mocht u hier vragen over hebben kunt u contact opnemen met de onderzoeker via onderzoek@ggdzl.nl.

**Uw gegevens worden vertrouwelijk behandeld**

De GGD voldoet aan de eisen die de privacywetgeving stelt. Uw ingevulde vragenlijstgegevens en consultgegevens worden door de datamanager van de GGD voorzien van een code om uw privacy te beschermen. Hiervoor heeft de datamanager kort inzage in uw e-mailadres. De datamanager heeft een geheimhoudingsverklaring getekend. Uw e-mailadres wordt verwijderd nadat de code is gekoppeld aan de vragenlijstgegevens. De vragenlijstgegevens worden gekoppeld aan de SOA consultgegevens op basis van de code. Bij het analyseren en rapporteren van het onderzoek zijn uw gegevens niet tot u te herleiden. Uw gegevens worden in een beveiligde opslagruimte bewaard voor de gangbare termijn van 15 jaar.

**Deelname is vrijwillig**

U beslist zelf of u meedoet aan het onderzoek. Als u niet wilt meedoen, zal dit op geen enkele manier invloed hebben op de zorg die u van de GGD ontvangt.

Bij vragen over het onderzoek kunt contact opnemen met de onderzoeker via [onderzoek@ggdzl.nl](mailto:onderzoek@ggdzl.nl)

Ik geef toestemming voor het verzamelen en gebruiken van mijn vragenlijstgegevens en mijn meest recente SOA consultgegevens voor de beantwoording van de onderzoeksvragen in dit onderzoek. Indien u geen toestemming geeft, kunt u helaas niet deelnemen aan het onderzoek.

- Ja
- Nee

Dear Sir,

We would like to ask you to participate in a study by the Public Health Service to improve our care and STI prevention. You are receiving this questionnaire because you indicated during your visit that we could invite you for this study. In this letter, you will be informed about what the research entails. Take your time to read this information. Should you still have any further questions after reading the information, the researcher will be glad to answer them.

**Background information**

The Public Health Service regularly receives questions from visitors about drugs. We want to improve our knowledge about this in order to provide care that meets the wishes and needs of visitors. Aims of this study are to:

- Investigate the prevalence of drug use during sex;

- Identify the context of drug use during sex;

- Investigate the association between drug use and STI;

- Identify needs for professional help.

Your participation is also important if you do not have any experience with using drugs.

**What does participation entail?**

Participating in this study means that you will complete a questionnaire that takes around 20 minutes. The answers to the questionnaire are only used for research purposes. The answers are not visible for STI nurses, doctor's assistants, or doctors. Participation does not affect the care you receive at the Public Health Service. The questionnaire data are linked to some data from the most recent STI consultation at the Public Health service, such as STI test results, postal code, ethnicity, age and level of education. If you have any questions about this, please contact the researcher via onderzoek@ggdzl.nl.

**Your data will be treated confidentially**

The Public Health Service meets the requirements set by the privacy legislation. A code will be linked to your completed questionnaire data and STI consultation data to protect your privacy. The data manager has brief access to your e-mail address to link the code to your data. The data manager has signed a confidentiality agreement. Your e-mail address will be deleted after the code is linked to your data. The encoded questionnaire data are linked to the consultation data based on the code. When analyzing and reporting research data, your data cannot be traced back to you. The data is stored in a secure storage room for the usual 15-year term.

**Participation is voluntary**

You decide yourself whether you will participate in this study. If you would rather not participate, this will in no way affect the care you receive from the Public Health Service.

If you have any questions about the research, please contact the researcher via [onderzoek@ggdzl.nl](mailto:onderzoek@ggdzl.nl)

I consent to the collection and use of my questionnaire data and my most recent STI consultation data for answering the research questions in this study. If you do not consent, you cannot participate in this study.

- Yes
- No
