# Supplementary material for: Attitude and beliefs about the social environment associated with chemsex among MSM visiting STI clinics in the Netherlands: An observational study
Source: PLoS One. 2020 Jul 1;15(7):e0235467. doi: 10.1371/journal.pone.0235467 (PMC7329118; doi:10.1371/journal.pone.0235467)
Supplement: S2 File — (DOCX) [file pone.0235467.s002.docx]

**S2 File List of participating STI clinics.**

- GGD Zuid-Limburg
- GGD Limburg Noord
- GGD Brabant Zuid-Oost
- GGD Hart voor Brabant
- GGD Gelderland-Midden
- GGD Gelderland-Zuid
- GGD Twente
- GGD Haaglanden
- GGD Ijsselland
