# Supplementary material for: Attitude and beliefs about the social environment associated with chemsex among MSM visiting STI clinics in the Netherlands: An observational study
Source: PLoS One. 2020 Jul 1;15(7):e0235467. doi: 10.1371/journal.pone.0235467 (PMC7329118; doi:10.1371/journal.pone.0235467)
Supplement: S3 File — (DOCX) [file pone.0235467.s003.docx]

**S3 File Online questionnaire in Dutch and English**

Questions in Dutch

1. De volgende vragen gaan over seks in het algemeen. In deze vragenlijst bedoelen we met seks elke vorm van seksuele activiteit: anale seks (kont), vaginale seks (vagina), orale seks (mond), manuele seks (handen) en seks met speeltjes.
2. Heb je seks gehad in de afgelopen zes maanden?

- Ja
- Nee

1. Welke seks heb je meestal? Je kunt één of meer antwoorden aankruisen.

- Aftrekken
- Ik pijpte hem
- Hij pijpte mij
- Zijn penis in mijn kont met condoom (anale seks ontvangend, bottom)
- Zijn penis in mijn kont zonder condoom (anale seks ontvangend, bottom)
- Mijn penis in zijn kont met condoom (anale seks gevend, top)
- Mijn penis in zijn kont zonder condoom (anale seks gevend, top)
- Ik likte zijn kont (rimmen)
- Zijn vuist in mijn kont (fisten)
- Speeltjes in mijn kont zonder condoom
- Ik weet het niet
- Anders, namelijk *Open*

1. Op welke plekken heb je meestal seks? Je kunt één of meer antwoorden aankruisen.

- Bij mij thuis
- Bij anderen thuis
- Op seksfeesten in Nederland
- Op seksfeesten in het buitenland
- Bij klanten (sekswerk)
- Darkroom
- Sauna
- Cruise area
- Op een after-party
- Anders, namelijk *Open*

1. Met hoeveel partners heb je meestal seks per keer?

- 1
- 2 of 3
- 4 of meer
- Meer dan 10
- Dat kan ik me niet herinneren

1. Met wie heb je meestal seks?

- Vaste partner (relatie)
- Vaste sekspartner(s) (fuck-buddy)
- Losse sekspartner(s) (je weet zijn naam)
- Groepsseks
- Klanten
- Vrienden
- Losse sekspartner(s) (je weet zijn naam niet)
- Vrouw
- Anders, namelijk *Open*

1. Hoe kom je meestal in contact met partners waarmee je seks hebt? Je kunt één of meer antwoorden aankruisen.

- Seksfeesten
- Sekswerk
- Ontmoetingsplekken, namelijk *Open*
- Online dating websites/apps, namelijk *Open*
- Andere social media, namelijk…
- Het is mijn vaste partner
- Op een andere manier, namelijk *Open*

1. Heb je ooit wel eens drugs gebruikt? Dit gebruik hoeft niet samen te hangen met seks, dit kan ook bijvoorbeeld tijdens een festival zijn geweest.

- Ja
- Nee *>> Vraag 17*

1. Welke drugs heb jij ooit gebruikt? Geef per drug aan of je deze ooit hebt gebruikt (ja of nee).

|  | **Ja** | **Nee** |
| --- | --- | --- |
| **Basecoke**, Crack |  |  |
| **Cannabis**, Hasj, Wiet, Marijuana |  |  |
| **Crystal meth**, Tina, Ice |  |  |
| **Cocaine**, Coke, Charlie, Blow, Snow, White |  |  |
| **GBL**, G, G-tje, Buisje, Tante Gea, Liquid Ecstasy |  |  |
| **GHB**, G, G-tje, Buisje, Tante Gea, Liquid Ecstasy |  |  |
| **Heroïne,** H, Smack, Chiba, Chiva, Bruin |  |  |
| **Ketamine,** K, Special K, Keta, Ket, Vitamin K |  |  |
| **Lachgas** |  |  |
| **LSD** |  |  |
| **Mephedrone,** Meow Meow, 4-MCC, M-Cat, Drone, Miaow, Plant food |  |  |
| **MDMA,** M, Molly |  |  |
| **MXE,** Mexxxy, Roflocptr |  |  |
| **Naphyrone,** NRG |  |  |
| **Paddo’s, truffels** |  |  |
| **Poppers** |  |  |
| **Ritalin, Concerta,** Dexamphetamine (niet als medicatie tegen ADD/ADHD) |  |  |
| **Speed,** Amphetamine, Pep |  |  |
| **XTC,** Ecstasy, Pil, Candy |  |  |
| **2-CB** |  |  |
| **3 MMC** |  |  |
| **4-FA,** 4-FMP, 4 Fluor, 4F, Flava |  |  |
| **Andere drugs** |  |  |

1. Je hebt net aangegeven dat je een andere drug hebt gebruikt dan in de tabel is aangegeven. Welke drug(s) zijn dit? Je kunt één, twee of drie andere namen van drugs invoeren.

- Naam andere drug: *Open*
- Naam andere drug: *Open*
- Naam andere drug: *Open*

1. Heb je wel eens drugs voor of tijdens seks gebruikt?
   - Ja
   - Nee >> Vraag 17
2. Hoe vaak heb je de volgende drugs gebruikt voor of tijdens seks in de afgelopen zes maanden? Geef per drug aan hoe vaak je deze hebt gebruikt.

*>> Drugs die zijn aangeklikt bij vraag 2 worden weergegeven.*

|  | 4 of meer keer per week | 2-3 keer per week | 2-4 keer per maand | 1 keer per maand of minder | Niet in de afgelopen zes maanden | Niet voor of tijdens seks |
| --- | --- | --- | --- | --- | --- | --- |
| Basecoke, Crack |  |  |  |  |  |  |
| Cannabis, Hasj, Wiet, Marijuana |  |  |  |  |  |  |
| Crystal meth, Tina, Ice, Crystal, T, Shabu, Yaba, Shista |  |  |  |  |  |  |
| Cocaïne, Coke, Charlie, Blow, Snow, White |  |  |  |  |  |  |
| GBL, G, G-tje, Buisje, Tante Gea, Liquid Ecstasy |  |  |  |  |  |  |
| GHB, G, G-tje, Buisje, Tante Gea, Liquid Ecstasy |  |  |  |  |  |  |
| Heroïne, H, Smack, Chiba, Chiva, Bruin |  |  |  |  |  |  |
| Ketamine, K, Special K, Keta, Ket, Vitamin K |  |  |  |  |  |  |
| Lachgas |  |  |  |  |  |  |
| LSD |  |  |  |  |  |  |
| Mephedrone, Meow Meow, 4-MCC, M-Cat, Drone, Miaow, Plant Food, MMC-4 |  |  |  |  |  |  |
| MDMA, M, Molly |  |  |  |  |  |  |
| MXE, Mexxxy, Rofloctptr |  |  |  |  |  |  |
| Naphyrone, NRG |  |  |  |  |  |  |
| Paddo‘s |  |  |  |  |  |  |
| Poppers |  |  |  |  |  |  |
| Ritalin, Concerta, Dexamfetamine (niet als medicijn voor ADD/ADHD) |  |  |  |  |  |  |
| Speed, Amfetamine, Pep |  |  |  |  |  |  |
| XTC, Ecstasy, X, Pill, Candy |  |  |  |  |  |  |
| 2-CB |  |  |  |  |  |  |
| 3 MMC |  |  |  |  |  |  |
| 4-FA, 4-FMP, Fluor, 4-F, F, Flava |  |  |  |  |  |  |
| 4-MEC |  |  |  |  |  |  |
| [{Drug1}] |  |  |  |  |  |  |
| [{Drug2}] |  |  |  |  |  |  |
| [{Drug3}] |  |  |  |  |  |  |

1. De volgende vragen gaan over het combineren van verschillende drugs. Met drugs combineren bedoelen wij verschillende drugs tegelijk of (vlak) na elkaar gebruiken.
2. Combineer je wel eens verschillende drugs voor of tijdens seks?

- Ja, geef aan welke combinatie je het meest gebruikt *Open*
- Nee

1. Hoe vaak heb je verschillende drugs gecombineerd voor of tijdens seks in de afgelopen zes maanden?

- 4 of meer keer per week
- 2-3 keer per week
- 2-4 keer per maand
- 1 keer per maand of minder
- Niet in de afgelopen zes maanden

1. Hoe heb je drugs genomen in de afgelopen zes maanden? Je kunt één of meer antwoorden aankruisen.

- Slikken (pil, drankje, bommetje (poeder in vloeitje))
- Laten smelten onder mijn tong
- Snuiven
- Roken
- Anaal (kont) inbrengen zonder spuit
- Spuiten in mijn kont zonder naald (booty bumping)
- Spuiten met een naald (slammen) door mijzelf of iemand anders
- Op een andere manier, namelijk *Open*

1. Hoeveel van je sekspartners gebruikt ook wel eens drugs voor of tijdens seks? Verschuif de punt op de balk.

Niemand – Iedereen.

1. Hoeveel van je vrienden of kennissen gebruikt wel eens drugs voor of tijdens seks? Verschuif de punt op de balk.

Niemand – Iedereen.

1. Heb je tabak gerookt in de afgelopen maand?

- Ja
- Nee

1. Hoe vaak heb je alcohol gedronken in de afgelopen zes maanden?

- 4 of meer keer per week
- 2 tot 3 keer per week
- 2 tot 4 keer per maand
- 1 keer per maand of minder
- Niet in de afgelopen zes maanden *>> Vraag 22*

1. Hoeveel alcoholische drankjes drink je normaal?

- 1 of 2
- 3 of 4
- 5 of 6
- 7,8 of 9
- 10 of meer

1. Geef aan in hoeverre je het eens bent met de volgende uitspraken.

|  | Helemaal mee oneens | Oneens | Neutraal | Eens | Helemaal mee eens |
| --- | --- | --- | --- | --- | --- |
| Ik loop meer risico op het krijgen van een SOA of HIV door het gebruiken van drugs tijdens seks |  |  |  |  |  |
| Ik denk dat ik meer kan genieten van seks als ik drugs gebruik |  |  |  |  |  |
| Ik denk dat mijn angst voor een SOA vermindert door het gebruiken van drugs tijdens seks |  |  |  |  |  |
| Mijn vrienden vinden drugsgebruik tijdens seks leuk |  |  |  |  |  |
| Ik ervaar soms druk van mijn vrienden of sekspartners om ook drugs tijdens seks te gebruiken |  |  |  |  |  |
| Ik weet hoe ik drugs kan weigeren tijdens seks als ik deze niet wil gebruiken |  |  |  |  |  |
| Het lukt mij om nee te zeggen tegen vrienden of sekspartners die me drugs tijdens seks aanbieden als ik deze niet wil gebruiken |  |  |  |  |  |
| Ik denk automatisch aan drugs wanneer ik seks heb |  |  |  |  |  |

Questions in English

1. The following questions relate to sex in general. In this questionnaire, sex is considered to be any kind of sexual activity: anal sex (butt), vaginal sex (vagina), oral sex (mouth), manual sex (hands) and sex with toys.
2. Did you have sex in the past six months?

- Yes
- No

1. What kind of sex do you normally have? Please select all that apply.

- Jerk off (wanking)
- I gave him a blow-job
- He gave me a blow-job
- His penis in my anus with a condom (receptive anal sex, bottom)
- His penis in my anus without a condom (receptive anal sex, bottom)
- My penis in his anus with a condom (insertive anal sex, top)
- My penis in his anus without a condom (insertive anal sex, top)
- I licked his anus (rimming)
- His fist in my anus (fisting)
- Toys in my anus without a condom
- I do not remember
- Other, please specify *Open*

1. At which places do you normally have sex? Please select all that apply.

- At my home
- At other people’s home
- Sex parties in the Netherlands
- Sex parties in cities abroad
- Customers
- Darkroom
- Sauna
- Cruise area (street, roadside service, park, beach)
- After-party
- Other, please specify *Open*

1. How many different steady male partners do you usually have at a time?

- 1
- 2 or 3
- 4 or more
- More than 10
- I cannot remember

1. With whom do you normally have sex? Please select all that apply.

- Regular partner (relationship)
- Regular sex partner (fuck-buddy)
- Casual sex partner(s) (you know their name)
- Group sex
- Customers
- Friends
- Casual sex partner(s) (you do not know their name)
- Woman
- Other, please specify *Open*

1. How do you normally contact sex partners? Please select all that apply.

- Sex parties
- Exchange sex
- Meeting place, please specify *Open*
- Online dating websites/apps, please specify *Open*
- Other social media, please specify *Open*
- Another way, please specify *Open*
- He is my partner (relationship)

1. Have you ever used drugs? This also includes recreational use, for example during a festival.

- Yes
- No *>> Question 17*

1. Which drugs have you ever used? Please indicate every drug you ever used (yes or no).

|  | **Yes** | **No** |
| --- | --- | --- |
| **Basecoke**, Crack |  |  |
| **Cannabis**, Hashish, Weed, Marijuana |  |  |
| **Crystal meth**, Tina, Ice |  |  |
| **Cocaine**, Coke, Charlie, Blow, Snow, White |  |  |
| **GBL**, G, Gina, Liquid Ecstasy |  |  |
| **GHB**, G, Gina, Liquid Ecstasy |  |  |
| **Heroin,** H, Smack, Chiba, Chiva, Brown |  |  |
| **Ketamine,** K, Special K, Keta, Ket, Vitamin K |  |  |
| **Laughing gas** |  |  |
| **LSD,** Acid |  |  |
| **Mephedrone,** Meow Meow, 4-MCC, M-Cat, Drone, Miaow, Plant food |  |  |
| **MDMA,** M, Molly |  |  |
| **MXE,** Mexxxy, Roflocptr |  |  |
| **Naphyrone,** NRG |  |  |
| **Magic mushrooms** |  |  |
| **Poppers** |  |  |
| **Ritalin, Concerta,** Dexamphetamine (not used as medication for ADHD/ADD) |  |  |
| **Speed,** Amphetamine, Pep |  |  |
| **XTC,** Ecstasy, Pil, Candy |  |  |
| **2-CB** |  |  |
| **3 MMC** |  |  |
| **4-FA,** 4-FMP, 4 Fluor, 4F, Flava |  |  |
| **Other drug(s)** |  |  |

1. You just indicated that you used other drug(s). Please write down the name of the other drug(s). The maximum number is 3.

- Name other drug: *Open*
- Name other drug: *Open*
- Name other drug: *Open*

1. Have you ever used drugs before or during sex?

- Yes
- No *>> Question 17*

1. How often have you used the following drugs before or during sex in the past six months?

*>>Drugs that are checked in question 2 are displayed.*

|  | 4 or more times per week | 2-3 times per week | 2-4 times per month | 1 time per month or less | Not in the past six months | Not before or during sex |
| --- | --- | --- | --- | --- | --- | --- |
| Basecoke, Crack |  |  |  |  |  |  |
| Cannabis, Hashish, Weed, Marijuana |  |  |  |  |  |  |
| Crystal meth, Tina, Ice, Crystal, T, Shabu, Yaba, Shista |  |  |  |  |  |  |
| Cocaine, Coke, Charlie, Blow, Snow, White |  |  |  |  |  |  |
| GBL, G, Gina, Liquid Ecstasy |  |  |  |  |  |  |
| GHB, G, Gina,, Liquid Ecstasy |  |  |  |  |  |  |
| Heroin, H, Smack, Chiba, Chiva, Bruin |  |  |  |  |  |  |
| Ketamine, K, Special K, Keta, Ket, Vitamin K |  |  |  |  |  |  |
| Laughing gas |  |  |  |  |  |  |
| LSD, Acid |  |  |  |  |  |  |
| Mephedrone, Meow Meow, 4-MCC, M-Cat, Drone, Miaow, Plant Food, MMC-4 |  |  |  |  |  |  |
| MDMA, M, Molly |  |  |  |  |  |  |
| MXE, Mexxxy, Rofloctptr |  |  |  |  |  |  |
| Naphyrone, NRG |  |  |  |  |  |  |
| Magic mushrooms |  |  |  |  |  |  |
| Poppers |  |  |  |  |  |  |
| Ritalin, Concerta, Dexamfetamine (no used as medication for ADHD/ADD) |  |  |  |  |  |  |
| Speed, Amfetamine, Pep |  |  |  |  |  |  |
| XTC, Ecstasy, X, Pill, Candy |  |  |  |  |  |  |
| 2-CB |  |  |  |  |  |  |
| 3 MMC |  |  |  |  |  |  |
| 4-FA, 4-FMP, Fluor, 4-F, F, Flava |  |  |  |  |  |  |
| 4-MEC |  |  |  |  |  |  |
| [{Drug1}] |  |  |  |  |  |  |
| [{Drug2}] |  |  |  |  |  |  |
| [{Drug3}] |  |  |  |  |  |  |

1. The next few questions will be about combining different drugs. By combining drugs we mean using different drugs simultaneously or (immediately) after each other.
2. Have you ever combined different drugs before or during sex?

- Yes, please indicate which combination you use most often *Open*
- No

1. How often have you combined different drugs before or during sex in the past six months?

- 4 or more times per week
- 2-3 times per week
- 2-4 keer per month
- 1 time per month or less
- Not in the past six months

1. How did you administer drugs or substances you used before or during sex in the past six months? Please select all that apply.

- Swallow (pill, liquid, bomb (powder in a paper))
- Melt under my tongue
- Snort/inhale
- Smoke
- Rectal administration without a syringe
- Rectal administration with a syringe without a needle (booty bumping)
- Inject (slamming) by myself or somebody else
- Other way, please specify *Open*

1. How many of your sex partners use drugs during sex? Slide the tip on the bar.

Nobody – Everybody.

1. How many of your friends use drugs during sex? Slide the tip on the bar.

Nobody – Everybody.

1. Have you smoked tobacco in the past month?

- Yes
- No

1. How often have you had a drink containing alcohol during the past six months?

- 4 or more times per week
- 2-3 times per week
- 2-4 times per month
- 1 time per month or less
- Not in the past six months *>>Question 22*

1. How many drinks containing alcohol do you have on a typical day when you are drinking?

- 1 or 2
- 3 or 4
- 5 or 6
- 7,8 or 9
- 10 or more

1. Please indicate to what extent you agree with the following statements.

|  | Totally disagree | Disagree | Neutral | Agree | Totally agree |
| --- | --- | --- | --- | --- | --- |
| I think that I am more at risk of acquiring an STI when I use drugs during sex |  |  |  |  |  |
| I think that I can enjoy sex more when I use drugs |  |  |  |  |  |
| I think that my fear of acquiring an STI decreases when I use drugs during sex |  |  |  |  |  |
| My friends consider using drugs during sex to be a fun thing to do |  |  |  |  |  |
| I sometimes experience pressure from my friends or sex partners to use drugs during sex |  |  |  |  |  |
| I know how to refuse drugs during sex if I do not want to use them |  |  |  |  |  |
| I manage to say no to friends or sex partners who offer me drugs during sex |  |  |  |  |  |
| I automatically think about drugs when having sex |  |  |  |  |  |
