# Supplementary material for: Attitude and beliefs about the social environment associated with chemsex among MSM visiting STI clinics in the Netherlands: An observational study
Source: PLoS One. 2020 Jul 1;15(7):e0235467. doi: 10.1371/journal.pone.0235467 (PMC7329118; doi:10.1371/journal.pone.0235467)
Supplement: Attachment — Submitted filename: Response to Reviewers file.docx [file pone.0235467.s004.docx]

Dear editor and reviewers,

Thank you for giving us the opportunity to revise our manuscript entitled: ‘Attitude and beliefs about the social environment associated with chemsex among MSM visiting STI clinics in the Netherlands: an observational study’.

We greatly thank the editor and reviewers for their helpful and constructive comments, all addressed below in our point-by-point reply. We revised and improved the manuscript accordingly. We are very pleased with the improvements made and hope you will find them satisfactory.

Kind regards, also on behalf of the co-authors,

Ymke Evers

**Journal requirements:**

1. Please ensure that your manuscript meets PLOS ONE's style requirements, including those for file naming. The PLOS ONE style templates can be found at

<https://journals.plos.org/plosone/s/file?id=wjVg/PLOSOne_formatting_sample_main_body.pdf> and

<https://journals.plos.org/plosone/s/file?id=ba62/PLOSOne_formatting_sample_title_authors_affiliations.pdf>

Response: We checked the style requirements and corrected sub-headings and references to supporting information that were not in line with the requirements.

2. Please refer to any sample size calculations or post-hoc corrections to correct for multiple comparisons during your statistical analyses. If these were not performed please justify the reasons. Please refer to our statistical reporting guidelines for assistance (<https://journals.plos.org/plosone/s/submission-guidelines.#loc-statistical-reporting>).

Response: As this was an exploratory study into various possible characteristics and beliefs associated with chemsex, we used convenience sampling for recruitment of participants. Our study is one of the first to assess associations between psychosocial beliefs (attitude, descriptive norm, etc.) and chemsex. We did not power the study a priori on a specific exposure and outcome of interest, as our exposure determinants were various and exploratory. Power of our resulting convenience sample (n=368) was sufficient (87%, assuming a R^2^ other X=0.45 as showed in Table 3, Step 3 before entering the psychosocial determinants in the final model) to detect odds ratios >1.5 at an alpha level of 5%.

We understand that multiple comparisons corrections, such as Bonferroni correction, are useful to decrease the probability of making a single type I error when comparing many simulated scenarios simultaneously. However, for exploratory studies, correcting multiple comparisons is often a too strict approach (leading to more type II errors) and a flexible approach is more suitable (Bender & Lange; Adjusting for multiple testing – when and how?; Journal of Clinical Epidemiology). As we did not aim to summarize our results into one conclusion for decision making, but aimed to describe characteristics and beliefs of MSM practicing chemsex to inform healthcare workers and researchers, we decided not to correct for multiple comparisons. We have now more clearly labeled our study as exploratory in the introduction, methods and discussion.

- In the introduction: ‘*Therefore, to explore characteristics and beliefs of MSM practicing chemsex, in the present quantitative study, we developed a comprehensive framework including psychosocial determinants, socio-demographics, tobacco and alcohol use, and sexual behaviour possibly associated with chemsex’*.
- In the methods: ‘*Convenience sampling was used’* and ‘*As this study concerns an exploratory study, we did not adjust for multiple comparisons’*.
- In the discussion: ‘*This is one of the first quantitative studies that assessed explored a broad set of psychosocial, sociodemographic and sexual behavioural determinants for its association with chemsex among MSM*.’
- *‘Finally, multiple comparisons might have increased risk for type 1 errors. Yet, for exploratory studies, correcting multiple comparisons is often a too strict approach (leading to more type II errors) and a flexible approach, as we took in our study, is deemed more suitable [37]’.*

3. Please provide additional details regarding participant consent. In the ethics statement in the Methods and online submission information, please ensure that you have specified how verbal consent was documented and witnessed.

Response: The following information is given in the ethics statement: ‘*This study was approved by the Medical Ethical Committee of the University of Maastricht (METC 2018-0485). STI client registry data was collected within standard care. During recruitment, STI nurses provided information on the study and participants gave oral informed consent to be approached for the online questionnaire and this was registered in the STI client registry. Participants gave written informed consent at the beginning of the online questionnaire (informed consent form can be found in S1 File). Data from questionnaires and linkage to data from medical records were retrieved in a coded de-identified manner’.*

4. Please include additional information regarding the survey or questionnaire used in the study and ensure that you have provided sufficient details that others could replicate the analyses. For instance, if you developed a questionnaire as part of this study and it is not under a copyright more restrictive than CC-BY, please include a copy, in both the original language and English, as Supporting Information. In addition, please include details of any pretesting of this questionnaire, including the number and nature of the participants.

Response: We have now included the online questionnaire in Dutch and English in the supporting information (S3 File).

5. We note that you have indicated that data from this study are available upon request. PLOS only allows data to be available upon request if there are legal or ethical restrictions on sharing data publicly. For information on unacceptable data access restrictions, please see <http://journals.plos.org/plosone/s/data-availability#loc-unacceptable-data-access-restrictions>

In your revised cover letter, please address the following prompts:

a) If there are ethical or legal restrictions on sharing a de-identified data set, please explain them in detail (e.g., data contain potentially identifying or sensitive patient information) and who has imposed them (e.g., an ethics committee). Please also provide contact information for a data access committee, ethics committee, or other institutional body to which data requests may be sent.

b) If there are no restrictions, please upload the minimal anonymized data set necessary to replicate your study findings as either Supporting Information files or to a stable, public repository and provide us with the relevant URLs, DOIs, or accession numbers. Please see http://www.bmj.com/content/340/bmj.c181.long for guidelines on how to de-identify and prepare clinical data for publication. For a list of acceptable repositories, please see http://journals.plos.org/plosone/s/data-availability#loc-recommended-repositories.

We will update your Data Availability statement on your behalf to reflect the information you provide.

Response: We have now updated our data availability statement: ‘*Data in this study contain potentially identifying sensitive patient information. Due to the General Data Protection Regulation, it is not allowed to distribute or share any personal data that can be traced back (direct or indirect) to an individual. Interested researchers are required to provide their name and institution to avoid misuse of this sensitive data and to align with the General Data Protection Regulation. Therefore, interested researchers may contact the head of the data-archiving of the Public Health Service South Limburg (Helen Sijstermans:* [*helen.sijstermans@ggdzl.nl*](mailto:helen.sijstermans@ggdzl.nl)*) when they would like to re-use data .*

6. We note that you have included the phrase “data not shown” in your manuscript. Unfortunately, this does not meet our data sharing requirements. PLOS does not permit references to inaccessible data. We require that authors provide all relevant data within the paper, Supporting Information files, or in an acceptable, public repository. Please add a citation to support this phrase or upload the data that corresponds with these findings to a stable repository (such as Figshare or Dryad) and provide and URLs, DOIs, or accession numbers that may be used to access these data. Or, if the data are not a core part of the research being presented in your study, we ask that you remove the phrase that refers to these data.

Response: As this was not a core part of this study, we removed this phrase.

7. Please include captions for your Supporting Information files at the end of your manuscript, and update any in-text citations to match accordingly. Please see our Supporting Information guidelines for more information: http://journals.plos.org/plosone/s/supporting-information.

Response: We have adjusted the in-text citations to match it with the captions of Supporting Information files.

**Reviewer #1:**

This paper addresses an important subject: the association between drug use for sexual pleasure and HIV infection among men who have sex with men. The paper is reasonably well written but there are a number of conceptual and analytical problems that would need to be addressed before consideration for publication. They are listed below.

Response: Thank you for your helpful and constructive comments.

1. Although the authors are correct that chemsex may involve a variety of substances, historically crystal meth has been identified and studied as the core and most problematic substance involved in chemsex. In the present study, 85% of MSM reports to use XTC or MDMA for chemsex, of which the short and long term effects are very different from crystal meth (reported only by 9%). The authors should describe these differences in terms of fysiology, psychosocial and psychopathological effects and risks for dependency. They should also keep it in mind when they discuss and interprete their findings. Since most other studies aim to explain and understand crystal meth use in relation to HIV infection and other sequelae, this may affect the comparability of the present study within the total body of literature on the subject.

Response: Thank you for this comment. We agree that effects and harms related to drug use might vary depending on the drugs used. We have now added the most used drugs in our study in the discussion and elaborated on information regarding possible differences in harms in the discussion:

*‘Chemsex was a prevalent behaviour (44% recent practice) in MSM who visited STI clinics in the Netherlands. XTC/MDMA, GHB/GBL and ketamine were the most used drugs during sex’.*

‘*In the long term, a study has shown that MSM practicing chemsex might be at risk of becoming drug dependent and not being able to return to enjoying sober sex. The risk of dependency might vary per drugs used. For example, crystal meth and GHB/GBL are relatively more likely to cause physical addiction than other drugs [31]’.*

Regarding the comparability of our study to other studies in the discussion, we referred to studies that not only assessed crystal meth but examined a variety of drugs used in a sexual context, including:

- Maxwell S, Shahmanesh M, Gafos M. Chemsex behaviours among men who have sex with men: A systematic review of the literature.
- A systematic review that included studies that examined numerous sex related drugs.
- Hegazi A, Lee MJ, Whittaker W, Green S, Simms R, Cutts R, et al. Chemsex and the city: sexualised substance use in gay bisexual and other men who have sex with men attending sexual health clinics.
- Included a wide variety of drugs, including mephedrone, GHB/GBL, crystal meth, cocaine, ketamine.
- Pufall EL, Kall M, Shahmanesh M, Nardone A, Gilson R, Delpech V, et al. Sexualized drug use ('chemsex') and high-risk sexual behaviours in HIV-positive men who have sex with men.
- Included crystal meth, GHB/GBL and mephedrone.
- Barrett P, O'Donnell K, Fitzgerald M, Schmidt AJ, Hickson F, Quinlan M, et al. Drug use among men who have sex with men in Ireland: Prevalence and associated factors from a national online survey.
- Included cannabis, cocaine, crystal meth, XTC/MDMA, mephedrone, GHB/GBL, ketamine, LSD, poppers, speed.

2. In their analysis, the authors merge group sex and "fisting" in one variable labeled "esoteric sexual practices". This variable should be disaggregated and factors reported and analyzed seperately. Group sex is not esoteric and one of the main features of chemsex and unlike "fisting" a strong independent predictor of HIV acquisition. It should not be lumped together with something of a complete different order. This reviewer also argues that group sex should not be seen/analyzed as a determinant of chemsex, but rather as an effect or something occurring downstream in the causal chain.

Response: We follow the reviewer’s suggestion and have now analyzed group sex and fisting as separate variables. We adjusted Table 2 and Table 3 accordingly.

We agree that these sexual risk behaviour variables could be effects of chemsex, as people under the influence of drugs might make more risky choices than were intended before the consumption of drugs. Nevertheless, group sex, fisting, multiple sex partners and having sex at the party scene could also be seen as determinants of chemsex. MSM have indicated to enjoy these sex events more when under the influence of drugs and men might meet other people who also use drugs at such events. Because of the cross-sectional design, we were unable to assess causal relationships between determinants and chemsex and this was described as a limitation in the discussion. For interpretation of these results we described the following in the discussion: ‘*Irrespective of causality, the presence of sexual health risks and possible drug and tobacco use related harms in MSM practicing chemsex confirms the importance of health promotion strategies aimed at minimizing these harms’.*

3. The authors define their psychosocial variable "habit" as "thinking about drugs when having sex". This is not how the habit forms itself. It is the other way around. When people think, fantasize or plan for sex, they think about drugs. Sex implies drugs and craving happens when thinking about it. Hence the thinking (and preparation) happens before and not during sex. It may be better to remove this variable from the model or clearly address its limitations in the methods or discussion section.

Response: We agree that the measurement of habit formation could benefit from a better formulation, such as ‘I automatically think about taking drugs when planning for/fantasizing about sex’. Nevertheless, we still think the current habit question gives an indication about having an automatic link between sex and drugs in our participants. We also think awareness of habitual chemsex behaviour is highly important for healthcare services to prevent future possible addiction problems.

We therefore decided to keep this variable in our analysis, but included the following in the limitations: ‘*Ideally, the psychosocial determinants (especially concerning habit formation) would have been measured by multiple validated items’.*

4. Non-response was considerable in the present study (53%). The authors should use data available from the electronic patient registry to compare responders with non-responders to assess potential bias and comment on the implications when generalizing and interpreting the results.

Response: We compared several available characteristics from the electronic patient registry of our participants with the total MSM population that visited the STI clinic during the study period to assess the generalizability of our results to all MSM visiting Dutch STI clinics. We described this comparison and implications in the discussion: ‘*The questionnaire was completed by high-risk MSM, because they were recruited at the STI clinic and participants relatively had more sex partners than the total MSM population (n=3493) who visited the participating STI clinics during the study period (median 6 vs. 5, p=0.02). This could have led to an overestimation of the proportion of MSM practicing chemsex but enabled us to assess determinants associated with chemsex. Furthermore, MSM with a younger age (median age 40 vs. 34 years, p<0.001) and a non-western ethnicity (7% vs. 18%, p<0.001) were underrepresented in our study’.*

**Reviewer #2**:

The study provides important findings about MSM practicing chemsex. A few comments for your consideration:

Response: Thank you for your helpful comments for improving our manuscript.

Major Comments:

1. The conclusion of “heterogeneous group regarding socio-demographics” is unlikely to be supported by the insignificant difference of socio-demographics between the two groups. Insignificant difference, from my point of view, simply shows that the background of the two groups are similar.

Response: Thank you for this comment. We have changed this information in the discussion into: ‘*Our study indicates that MSM practicing chemsex are a heterogeneous group, as chemsex was comparably present in MSM of different age groups, socioeconomic statuses, ethnicities, educational levels and in MSM living in urban and non-urban areas’.*

2. In DISCUSSION, paragraph 2, “the clustering of sexual health risks and possible drug and tobacco use” is unlikely to be supported by the findings. No cluster analysis was performed in this study. Please consider if you want to modify the description.

Response: We agree that this term could introduce confusion. We have changed the description: ‘*Irrespective of causality, the presence of sexual health risks and possible drug and tobacco use related harms in MSM practicing chemsex confirms the importance of health promotion strategies aimed at minimizing these harms’*.

Minor Comments:

3. ABSTRACT line 30, please indicate clearly that the study population referred to those having sex in the past 6 months.

Response: We have added the following in the abstract to make this clear: ‘*In 2018, 785 MSM were recruited at eight Dutch STI clinics; 368 (47%) fully completed the online questionnaire. All participants reported to have had sex in the past six months’.*

4. INTRODUCTION lines 97-98: please move Figure 1 (and its citation) to METHODS

Response: We think that this figure better fits in the introduction, when describing the literature, on which the concepts in the framework are based.

5. METHODS: Different in-text citation methods are observed, please standardise, e.g. line 111 compared with lines 134-135

Response: Thank you for making us aware of these wrong format of citations. We have changed the citations in lines 111 and 134-135 into the correct format.

6. References in lines 178-179 are missing

Response: We have added the following in this sentence: ‘*For each block, the explained variance was described. The order of block-entry was based on previous literature (as described in Fig 1), known categories of variables associated with chemsex were entered first and the new category of variables, i.e. psychosocial determinants, were entered in the final step’.*

7. RESULTS: I am wondering if there are any multidrug data

Response: We added the following information about multiple drug use in the results: ‘*The use of four or more different drugs in the past six months was reported by 42% of MSM practicing chemsex. Combining different drugs during one chemsex session was reported by 81%; XTC/MDMA and GHB/GBL was the most reported combination (75%)’.*

8. Mismatch between RESULTS and TABLE 2. In main text, the psychological determinants are described in terms of median, while TABALE 2 shows mean and SD. Interestingly, the number is the same. Please clarify whether they are median and IQR or mean and SD.

Response: Thank you for this comment. In main text, means were meant instead of medians. In the Table, also means and standard deviations were reported. We changed the main text to make this consistent: ‘*MSM who reported chemsex more often believed that they could enjoy sex more when using drugs (mean attitude 3.9 vs. 2.2, p<0.001), felt less skilled to refuse drugs (mean refusal skills: 4.5 vs. 4.6, p=0.02), perceived greater acceptance (mean subjective norm: 3.7 vs. 2.8, p<0.001) and performance (mean descriptive norm: 3.7 vs. 2.8, p<0.001) of chemsex among their social environment, and scored higher on automatically thinking about drugs when having sex (mean habitual thought: 2.2 vs. 1.3, p<0.001) compared to MSM who did not report chemsex. Table 2 shows all other variables that were significantly associated with chemsex in univariable analyses’.*
